# Perceived Relevance of Educative Information on Public (Skin) Health: Results of a Representative, Population-Based Telephone Survey

**DOI:** 10.3390/ijerph121114260

**Published:** 2015-11-09

**Authors:** Daniela Haluza, Markus Schwab, Stana Simic, Renate Cervinka, Hanns Moshammer

**Affiliations:** 1 Institute of Environmental Health, Center for Public Health, Medical University of Vienna, Kinderspitalgasse 15, A-1090 Vienna, Austria; E-Mails: markus.schwab2@gmx.at (M.S.); renate.cervinka@meduniwien.ac.at (R.C.); hanns.moshammer@meduniwien.ac.at (H.M.); 2 Institute of Meteorology, University of Natural Resources and Life Sciences, Vienna, Peter-Jordan-Straße 82, A-1190 Vienna, Austria; E-Mail: stana.simic@boku.ac.at

**Keywords:** melanoma prevention, skin health promotion, sun protection, Public (Skin) Health, gender, health education

## Abstract

Individual skin health attitudes are influenced by various factors, including public education campaigns, mass media, family, and friends. Evidence-based, educative information materials assist communication and decision-making in doctor-patient interactions. The present study aims at assessing the prevailing use of skin health information material and sources and their impact on skin health knowledge, motives to tan, and sun protection. We conducted a questionnaire survey among a representative sample of Austrian residents. Print media and television were perceived as the two most relevant sources for skin health information, whereas the source physician was ranked third. Picking the information source physician increased participants’ skin health knowledge (*p* = 0.025) and sun-protective behavior (*p* < 0.001). The study results highlight the demand for targeted health messages to attain lifestyle changes towards photo-protective habits. Providing resources that encourage pro-active counseling in every-day doctor-patient communication could increase skin health knowledge and sun-protective behavior, and thus, curb the rise in skin cancer incidence rates.

## 1. Introduction

Exposure to ultraviolet radiation (UVR) is associated with acute skin reactions such as sunburn and tanning, as well as chronic skin damage including various forms of skin cancer [1]. These photo-induced skin manifestations are preventable by motivating individuals to diminish lifestyle-associated risk factors and boost photo-protective measures [2,3]. Also, preventive efforts reduce mid- and long-term costs for medical treatment of all types of respective dermatological lesions [4]. In spite of skin health education campaigns, epidemiologists report increasing incidence and mortality rates of cutaneous malignances, including melanoma and non-melanoma skin cancers worldwide [5,6,7]. As a possible explanation for this alarming trend, the prevailing common perception that a tanned skin is attractive could elicit recreational tanning activities [8]. Still, public concerns regarding potential health risks of artificial and natural UVR exposure are underestimated [9,10].

Individual skin health perception and knowledge are influenced by various factors including advertising, electronic information sources, mass media, family, and friends [11]. Evidence-based information materials also support doctor-patient communication and decision-making [12]. Skin health knowledge and preventive behavior differ significantly among countries, potentially due to varying national skin information strategies [13,14]. So, population-specific documentation facilitates the development and assessment of national Public Health campaigns. Traditionally, abundant scientific evidence is available from skin health research conducted in Australia and Northern European countries like Sweden and Denmark [15,16,17,18].

Austria is an alpine, German-speaking country in Central Europe. The rising skin cancer incidence rates among the mainly fair-skinned Austrian population are caused by high ambient UVR levels in the mountainous regions, but also leisure-time sun exposure [19]. So far, little is known about prevailing skin health attitudes and behavior to estimate the impact of primary prevention measures on reducing skin cancer incidence rates. To close this knowledge gap, we conducted consecutive scientific research projects subsumed under the umbrella term Public (Skin) Health [19,20,21,22,23]. This concept refers to evaluation of public and private measures to prevent skin diseases, promote skin health, and monitor populations at risk. Given the known publishing source bias found in information material available in Austria, the current population-based study assessed the influence of perceived relevance of specific skin health information material and sources on individual sun protection and tanning behavior among Austrian citizens [20].

There is empirical evidence that subjects with adequate skin health knowledge and sun protection use are more likely receiving respective information from healthcare professionals [22,24]. Thus, as a second objective, we studied the hypothesis whether or not the perceived relevance of the physician as a source of skin health information had impact on individual levels of recreational sun-protection practices, skin health knowledge, and motives to tan. Gender is the most recurrently reported socio-economic feature influencing skin health behavior [21,23,25,26,27]. Likewise, age-adjusted skin cancer incidence and mortality rates differ between females and males in Austria and also worldwide [19,28,29]. Accordingly, we investigated gender-specificity of prevailing use and acceptance of information material and sources.

## 2. Methods

### 2.1. Data Collection

This study was embedded in the larger interdisciplinary *UVSkinRisk* research project and presents findings gathered in a representative population-based telephone survey [19,21,27]. The study design was approved by the local ethical committee of the Medical University of Vienna and performed following the ethical guidelines and principles of the International Declaration of Helsinki. We contracted the market research company Triconsult based in Vienna as a third party to provide quality control and ensure respondents' anonymity. Data were collected in August 2011 among adult Austrian residents aged between 18 and 74 years. The gender-balanced study sample representing the Austrian population by means of age and place of residence were draw from the official national telephone directory list comprised a predetermined number of 1500 completed interviews. Survey questionnaire design and software assistance prevented missing data due to partial response or item nonresponse. All study subjects provided oral informed consent; participation in the telephone survey was voluntarily and confidential.

### 2.2. Study Questionnaire

The structured, computerized questionnaire was based on the study questionnaire in German established in a previously conducted paper-pencil survey [22,23]. We assessed demographic data (age, gender, educational level, smoking habits), self-reported skin type ranging from fair (I) to dark (VI) skin, occurrence of sunburns, skin health knowledge as well as behavioral and motivational factors that are known to influence personal photo-protective habits [30].

A quiz comprising of seven true-false questions tested participants’ knowledge of important skin health facts including UVR exposure, skin cancer, and sun protection. The composite score “knowledge score” summed up correct responses to true-false questions ranging from weak knowledge (=0) to full knowledge (=7). A set of items assessed frequency of eight sun-protective measures, namely “For sun protection I use sunscreen (min. SPF 15)/reapply sunscreen during the day/reapply sunscreen after swimming/avoid midday sun/seek shade/wear a hat/wear protective garments/wear sunglasses.” Following the grading system used in Austrian schools, the according five-point Likert scale scored from “always” (=1) to “never” (=5). Participants were asked to rate their degree of agreement with eight statements related to motives to tan, namely “A tanned skin is desirable because it enhances sex appeal/enhances attractiveness/enhances self-confidence/enhances fitness/enhances body shape/reduces paleness/reduces acne/reduces stretch marks.” using a five-point Likert scale ranging from “strongly agree” (=1) to “strongly disagree” (=5). We calculated mean response scores of the scales showing acceptable internal consistencies with Cronbach’s alpha (α) = 0.64 for the covariate “motives to tan” and α = 0.73 for the covariate “sun protection”. Subsequent median splitting dichotomized the knowledge score and the scales motives to tan and sun protection at cutoff point of ≤ or > than the median (low/high).

Further, participants indicated predominantly used educative material by answering the multiple-answer question “From where do you get your knowledge about sun protection?” offering the three choices healthcare providers, sunscreen producers, and tanning parlors. The multiple-response question “Which of the following sources of information about sun protection are relevant to you?” asked for perceived relevance of the eight information sources print media, television, family, physician, Internet, friends, radio, and school.

### 2.3. Statistical Data Analysis

We reported the obtained results as proportions, means, and standard deviation (SD) values where appropriate. Univariate descriptive analysis (Chi^2^ tests) evaluated gender differences regarding perceived relevance of information material and sources. One-way analysis of variance (Anova) investigated effects of amount of information sources and also the source physician *vs.* other sources on skin health knowledge, motives, and sun-protective behavior. Multiple logistic regression analyses evaluated the impact of picking the source physician on contextual and skin health-related characteristics. We performed both crude and adjusted (multiple) regression models. Cox and Snell R^2^, Nagelkerke’s R^2^, Pearson’s Chi^2^, and the Hosmer-Leme show goodness-of-fit tests assessed overall model performance and internal calibration. We reported the adjusted odds ratios (OR), 95% confidence intervals (CI), and *p*-values of the best-fitting model. We statistically processed the collected data using Excel spreadsheet (Microsoft, Redmond, WA, USA) and SPSS Version 22.0 (IBM Corp., New York, NY, USA). For all statistical analyses, a result was considered significant at the 5% critical level (*p* < 0.05).

## 3. Results

Table 1 depicts basic data on the study population (*n* = 1500, 49.5% males). Respondents were aged between 18 and 74 years (mean = 44.7 years, SD = 15.4, females: mean = 45.8, SD = 15.5, males: mean = 43.6, SD = 15.4), also see [19,24]. Most participants were educated to secondary education (47%), were non-smokers (56%), had skin type III (44%, all: gender differences *p* < 0.001), and lived in a relationship (66%). Male and female study subjects achieved similar results in the knowledge test (overall mean = 4.3, SD = 1.1) and amount of agreement with motives to tan (overall: mean = 3.8, SD = 0.9).

Further, we ranked amount of picked information material and sources overall and stratified by gender. As shown in Table 2, the top-ranked information material was healthcare providers (83%), whereas print media (57%), television (39%), and physician (38%) were the three top-ranked information sources. We found similar results for the rankings performed by females and males, with slight differences only for the sources family and radio. We revealed statistically significant gender differences for information material issued by healthcare providers (*p* = 0.014) and the information source print media (*p* = 0.019), both of which were picked more often by female participants.

We divided participants into two subgroups according to reporting few *vs.* numerous sources and picking the source physician *vs.* other sources. Additionally, we assessed respondents’ perceived relevance of information sources indicated by amount of picked sources (mean = 2.2, SD = 1.4, males: mean = 2.2, SD = 1.4, females: mean = 2.3, SD = 1.4, *p* = n.s.) and materials (mean = 1.5, SD = 0.6, males: mean = 1.4, SD = 0.6, females: mean = 1.5, SD = 0.6, *p* = 0.013). As shown in Table 3, amount (few *vs.* numerous) and specificity (picking the information source physician *vs.* all other sources) of information sources statistically significantly differed regarding sun protection (*p* = 0.001).

**Table 1:** Basic characteristics of study population, stratified by gender.

Factors			Gender				*p*-Value
	Overall		Females (*n* = 758)		Males (*n* = 742)		
	*n*	%	*n*	%	*n*	%	
**Age; years**							
18–29	305	20.3	145	19.1	160	21.6	0.243
30–39	278	18.5	135	17.8	143	19.3	
40–49	340	22.7	167	22.0	173	23.3	
50–59	260	17.3	134	17.7	126	17.0	
60–74	317	21.1	177	23.4	140	18.9	
**Educational level**							
Primary	357	23.8	189	24.9	168	22.6	0.001 **
Secondary	706	47.1	386	50.9	320	43.1	
Tertiary	437	29.1	183	24.1	254	34.2	
**Living situation**							
Single	507	33.8	244	32.2	263	35.4	0.183
Partner	993	66.2	514	67.8	479	64.6	
**Smoking habits**							
Smoking	346	23.1	145	19.1	201	27.1	0.001 **
Ex-smoking	313	20.9	135	17.8	178	24.0	
Non-smoking	841	56.1	478	63.1	363	48.9	
**Skin type**							
I	79	5.3	54	7.1	25	3.4	0.001 **
II	441	29.4	223	29.4	218	29.4	
III	657	43.8	340	44.9	317	42.7	
IV–VI	323	21.5	141	18.6	182	24.5	
**Knowledge score**							
Low	319	21.3	161	21.2	158	21.3	0.980
High	1181	78.7	597	78.8	584	78.7	
**Motives to tan**							
Low	732	48.8	388	51.2	344	46.4	0.062
High	768	51.2	370	48.8	398	53.6	
**Sun protection**							
Low	722	48.1	417	55.0	305	41.1	0.001 **
High	778	51.9	341	45.0	437	58.9	

**, *p* < 0.001; Chi^2^ test for gender differences.

**Table 2:** Amount of picked skin health information material and sources, stratified by gender and ordered by total rank.

Information Medium	Overall		Gender					Rank Overall (Females/Males)
			Females (*n* = 758)		Males (*n* = 742)		*p*-Value	
	*n*	%	*n*	%	*n*	%		
**Information material ^1^**								
Healthcare providers	1247	83.1	648	85.5	599	80.7	0.014*	1 (1/1)
Sunscreen producers	804	53.6	410	54.1	394	53.1	0.751	2 (2/2)
Tanning parlors	160	10.7	86	11.3	74	10.0	0.388	3 (3/3)
**Information sources ^2^**								
Print media	854	56.9	454	59.9	400	53.9	0.019 *	1 (1/1)
Television	585	39.0	310	40.9	275	37.1	0.128	2 (2/2)
Physician	567	37.8	300	39.6	267	36.0	0.151	3 (3/3)
Internet	336	22.4	169	22.3	167	22.5	0.922	4 (4/4)
Family	274	18.3	130	17.2	144	19.4	0.258	5 (6/5)
Radio	272	18.1	147	19.4	125	16.8	0.201	6 (5/6)
Friends	139	9.3	73	9.6	66	8.9	0.623	7 (7/7)
School	91	6.1	64	6.1	45	6.1	0.997	8 (8/8)

^1^ “From where do you get your knowledge about sun protection?”; ^2^ “Which of the following sources of information about sun protection are relevant to you?”; Values are presented as *n* (%); Chi^2^ test for gender differences; * *p* < 0.05.

**Table 3:** Results from Anova for effects of information sources on skin health knowledge, motives to tan, and sun protection.

Information Sources	Dependent Variable	df1	Mean1	df2	Mean2	F	*p*-Value
Few *vs.* numerous sources	Knowledge score	1	4.31 (1.14)	1498	4.27 (1.14)	0.22	0.639
	Motives to tan	1	3.82 (0.89)	1498	3.81 (0.81)	0.35	0.859
	Sun protection	1	14.23 (6.04)	1498	15.61 (6.60)	10.44	0.001 *
Physician *vs.* other sources	Knowledge score	1	4.35 (1.09)	1498	4.27 (1.17)	1.43	0.223
	Motives to tan	1	3.81 (0.89)	1498	3.83 (0.87)	0.25	0.620
	Sun protection	1	16.04 (5.51)	1498	13.50 (6.10)	66.31	0.001 *

df = degrees of freedom, for two-group design: df1 = 1, df2 = df residuals. * *p* < 0.001.

In sum, ranking of information material and sources revealed only slight differences in gender-specific ranking, *i.e.*, for the sources family and radio (Table 2), and amount of material and also having picked physician *vs.* other sources statistically significantly influenced sun-protective behavior (Table 3). Thus, the physician was the most powerful influencing factor for skin health protection when compared to all other information sources. To further evaluate respective predictors of perceiving the source physician as relevant skin health information sources (dependant variable), we conducted a multiple regression analysis. Due to their known influence on skin health-related knowledge and attitudes, we defined these factors as independent variables: Contextual characteristics including gender (females *vs.* males), age groups (in years, younger *vs.* older ages), educational level (tertiary *vs.* lower education), sunburns (no *vs.* yes), knowledge score (low *vs.* high) as well as health behavior including smoking habits (smokers *vs.* non/ex-smokers), sports activity and sunbed use (both: no *vs.* yes), sunbathing days (>15 days *vs.* fewer days), motives to tan, and sun protection (both: low *vs.* high) [11,13,21,23,25,26,27]. The Hosmer-Lemeshow goodness-of-fit statistic (Chi^2^ = 9.4, *p* = 0.307) indicated a good calibration of our best-fitting regression model with overall good performance (Cox and Snell R^2^ = 0.046, Nagelkerke’s *R*^2^ = 0.063, *p* < 0.001).

As shown in Table 4, independent predictors were older age, higher education level, fewer sunburns, reporting sport activity, and higher sun protection. In detail, compared to the youngest age group (18–30 years), participants older than 51 years (OR = 1.6, 95% CI 1.1–2.3) and sportspersons (OR = 1.4, 95% CI 1.1–1.8) were more likely to pick the information source physician, whereas participants educated to a lower educational level were less likely (OR = 0.7, 95% CI 0.5–1.0). Odds for reporting sunburn occurrence during the preceding year were lower among those receiving skin health information from a physician (OR = 0.7, 95% CI 0.6–0.9), all *p* < 0.05. This corresponds to the finding that performing low photoprotection was less likely when the physician was perceived as a relevant skin health information source (OR = 0.5, 95% CI 0.4–0.7, *p* < 0.001).

**Table 4:** Logistic regression analysis of factors affecting the prevalence of picking the information source physician. Odds ratio (OR), 95% confidence interval (95% CI), and overall factor-specific *p*-values are depicted.

Factors	*n*	%	OR (95% CI)	*p*-Value
Total	1500	100		
Information source physician (yes)	567	37.8		
**Contextual characteristics**				
Gender (female)	300	20.0	1.0 (0.8–1.3)	0.681
Age; years				0.028 *
18–30	93	6.2	1.0 = Ref	-
31–50	237	15.8	1.2 (0.9–1.7)	0.174
>51	237	15.8	1.6 (1.1–2.3)	0.004 *
Educational level				0.012 *
Primary	116	7.7	0.7 (0.5–1.0)	0.026 *
Secondary	279	18.6	1.0 (0.8–1.3)	0.992
Tertiary	172	11.5	1.0 = Ref	-
Sunburn 2010 (yes)	147	9.8	0.7 (0.6–0.9)	0.008 *
Knowledge score (high)	453	30.2	1.1 (0.9–1.3)	0.500
**Health behavior**				
Smoking habits				0.291
Smoker	331	22.1	1.0 = Ref	-
Ex-smoker	110	7.3	1.0 (0.7–1.2)	0.720
Non-smoker	126	8.4	0.8 (0.6–1.1)	0.239
Sport activity (yes)	385	25.7	1.4 (1.1–1.8)	0.0001 *
Sunbed use (yes)	45	3.0	0.9 (0.6–1.3)	0.471
Sunbathing; days				0.888
Never	191	12.7	0.9 (0.7–1.2)	0.494
<15	219	14.6	0.9 (0.7–1.2)	0.527
>15	157	10.5	1.0 = Ref	-
Motives to tan (low)	277	18.5	1.1 (0.9–1.4)	0.497
Sun protection (low)	827	55.1	0.5 (0.4–0.7)	0.0001 **

* *p* < 0.05, ** *p* < 0.001.

## 4. Discussion

The present cross-sectional study provides so far lacking empirical insight into prevailing use of skin health information media in association with lifestyle habits influencing recreational outdoor UVR exposure among a representative sample of the Austrian population. In addition, we analyzed whether medical counseling on skin health promotion effectively influenced sun exposure decisions. Study results and potential implications for practice, theory, and methodology are discussed from a Public (Skin) Health perspective.

Skin health beliefs are influenced by diverse factor including family, friends, and mass media, as suggested by Goulart and co-workers [31]. Regarding Internet-based information, online educative websites are cheap and time-independently accessible tools for communicating health messages [32]. Moreover, printed educative material could influence health decisions and doctor-patient relationship by empowering patients [33]. Ranking of information material and sources revealed that study subjects relied on the more traditional media magazines and television ahead of Internet for skin health advice. Lagging behind traditional media, the source physician was ranked third. We found that amount of material as well as picking the physician compared to all other information sources as a measure for received skin health advice by a medical professional statistically significantly influenced sun-protective habits of participants. Besides this, regression analysis revealed that fewer sunburns as well as higher sun protection predicted relevance of the information source physician. Thus, our study identified the physician as the most powerful influencing factor for skin health promotion. This observation is in line with previous publications reporting that subjects with more skin health knowledge and sun protection mostly received information from healthcare providers [22,24]. This finding might give rise to optimism regarding contextually practiced national lifestyle counseling and narrative medicine, which is usually perceived as a time- and resource-consuming task.

Encouraging photo-protection, especially regular sunscreen use, was identified to be the central health message in preventative skin health counseling [18]. Magdum and co-workers identified dermatologists and plastic surgeons as principal stakeholders in skin cancer treatment, as such standing at the forefront of influencing sun protection measures [34]. In line with Bragazzi *et al.*, our data suggest that healthcare professionals including non-dermatologist physicians should pro-actively activate their patients to adopt adequate sun-protective measures [35]. To distribute health promotion messages, representatives of all medical disciplines and also medical students should internalize the economic and health benefits of primary prevention [34]. Healthcare staff should be trained to empathically explain how lifestyle may affect prospect skin health by communicating with each individual in a way that maximises understanding and promotes a positive attitude toward health maintenance. Occupational regulations and professional policies of competent medical authorities could promote respective evidence-based counseling by recommending consultation practices in (continuing) medical education and specialist training regulations.

Tanning as the skin’s response to UVR exposure is associated with epidermal cell damage and potential skin cancer risk [1,36]. Despite of public awareness campaigns, a tanned skin is still desirable and connected to positive appearance in Western societies. Thus, the perceived social value of a sun tan might over-ride individual’s skin health actions if these contradict peer activities and positive attitudes towards intentional tanning, time spent in the sun, and vacations to sunny resorts [16]. In Australia, health campaigns effectively motivated behavioral changes among sunbathers and even reduced skin cancer rates [17,18,37]. Thus, these mass media campaigns raised awareness for skin health promotion and advantageously influence tanning-related attitudes. Conflictingly, increased sun-related knowledge does not change sun-related behavior, suggesting a need for repeatedly provided educative content [21,23,35,38]. Also, future skin health campaigns might be more effective if they target social and psychological barriers associated with non-uptake of sun protection. In line with these study results, we suggest that evaluating country and target group-specific barriers could unravel the complex factors contributing to translate knowledge into skin health action [35].

In this survey, study subjects perceived that healthcare providers were the most important publishing issue of respective material. Nevertheless, participants were proportionally often also familiar with covert advertising-prone information material distributed by sunscreen producers and tanning parlors [20,22]. Information material published by healthcare providers aim at preventing negative health effects of sun bathing, whereas that by sunscreen producers clearly seek to primarily activate potential consumers to buy sunscreen products. Employees of tanning parlors usually provide information material stressing advantages of having a tanned skin and do not educate on potential health hazards of artificial UVR exposure. These findings claim for the need to monitor contents of these material to reduce the obvious publishing source bias in information material on medical issues [20].

Internet-based, interactive, educational programs could enhance public participation in skin cancer prevention [39]. Trinh *et al.* showed that audio-visual presentation compared to printed information material was more effective in educating on sun-protective behaviors among transplant patients [40]. As web-based information could lack completeness and accuracy, healthcare professionals should raise awareness on variable educative quality and thus, pro-actively recommend comprehensive Web-based health information material [41].

Recent advances in communication technology represent a brave new world for innovative healthcare initiatives [42,43]. Increasingly, physicians adopt this new technology to distribute health information and interact with patients and the healthcare community. Also, the power of social networking tocost-effectively advertise commercial products as well as societal norms increasingly attracts attention. However, social media also introduce risks for information accuracy, organizational reputation, and individual privacy. U.S. licensing authorities have already reported several breaches of medical professionalism resulting in disciplinary actions [44]. According to Chauhan *et al.*, misuse of physician’s freedom of speech via social media channels and Internet blogs can reach millions instantly and cause irreversible harm due to misinformation, warranting a taskforce to identify potential harmful postings [44]. Still, European-wide regulations are missing to monitor practices and quality of information to address the problems accompanied with publishing bias and sprawling of freedom of speech.

The mass media’s powerful influence can directly affect public opinions and consumerism. However, mainstream media might not be suitable to unambiguously provide balanced educative content to consumers who tend to predominantly receive information passively [45]. Attention-grabbing headlines do not reflect the cutting edge of scientific knowledge, inflicting the need for monitoring printed and online media [46]. Portraying tanned celebrities in magazines and television programmes contradicts public health campaigns by glorifying a tanned skin [45]. Consumers might be motivated to copy physical appearances of these fashion and style role models to comply with this perceived aspirational standard [11]. In this context, target group-sensitive skin health information should focus on disadvantageous outcomes on appearance of sun exposure and inadequate skin protection [47]. Also, avoiding prohibitions in favour of affirmatively phrased health messages might create a favourable societal atmosphere. Adequate governmental funding is needed for financing collaborations to assist media-producing agencies in providing graspable formats for respective educative contents [48].

Quality criteria for evidence-based patient information material including the use of patient narratives as well as clear drawings and pictures to enhance knowledge und comprehension have already been suggested [48]. Although Austrian information materials have been shown to be poor in quality and present contradictory or incomplete evidence, a major thread throughout educative folders for Public (Skin) Health promotion and other preventive health topics in Austria is still lacking [20,49]. For educating on health risks, effectiveness and persuasiveness of gain- and loss-framed prevention messages and language based features (high *vs.* low-intensity language) are yet unknown for Austrian conditions [48,50,51]. The current study reports that factors predicting gathering skin health information from the source physician - besides fewer sunburns and more sun protection - were older age, higher education level, and sport activity. These findings could stimulate future research and discussions among healthcare stakeholders on which outline would be the most appropriate to target group-specifically inform the public on the advantages and disadvantages of UVR exposure.

### Limitations

As this analysis was based on a large, population-based, nationally representative Austrian study sample, we assume that our data represent a trustworthy picture of the actual relevance of skin health information media and recreational habits executed by the Austrian population. Nevertheless, study limitations included possibility of reporting and recall bias due to subjectivity of the self-reported outcomes. However, previous studies indicated that self-reported sun protection behaviors were well correlated with UV dosimeter readings and direct observation [38]. The type and quality of received skin health information, health seeking behavior, and consultation by general practitioners or specialized doctors could not be ascertained from the data. Also, we used cross-sectional data, which could not bring out the direction of causality between the use of information sources and material with self-reported behavioral, attitudinal and motivational factors. I line with the literature, we thus assumed that questionnaire items assessing sun exposure-related behaviors and attitudes were stable and reliable [52,53].

Notwithstanding these limitations, the paper added so far lacking scientific evidence on prevailingly used communication media types and identified potential key factors associated with skin health behavior in Austria. We believe that the current research has wide implications for clinical practice and community prevention and these findings could be valuable for medical professionals and healthcare stakeholders responsible for skin health-related advocacy, funding, and authorization decisions.

## 5. Conclusions

The present study suggests that although the physician is not rated as the most relevant channel for skin health information, medical counseling serves as reliable and effective means of modifying risky sun exposure. From a public (skin) health perspective, evidence-based, target group-specific health information should strive at primary prevention of UVR-associated skin diseases, reducing human suffering and healthcare expenses. On a societal level, incentive and reward schemes could motivate individuals to care for health and well-being of themselves and their next of kin, and by doing so, additionally serveas encouraging role model for children and adolescents.
